# The regulatory mechanisms controlling meiotic cross-over patterning in plants

**DOI:** 10.1042/BST20253025

**Published:** 2025-09-12

**Authors:** Wanyue Xu, Qichao Lian, Meiling Li, Gregory P. Copenhaver, Yingxiang Wang

**Affiliations:** 1 State Key Laboratory of Genetic Engineering, Institute of Plant Biology, School of Life Sciences, Fudan University, Shanghai, 200433, China; 2 Guangdong Laboratory for Lingnan Modern Agriculture, Guangdong Provincial Key Laboratory for the Development Biology and Environmental Adaptation of Agricultural Organisms, College of Life Sciences, South China Agricultural University, Guangzhou, 510642, China; 3 Department of Biology and the Integrative Program for Biological and Genome Sciences, University of North Carolina at Chapel Hill, Chapel Hill, NC, 27599-3280, USA; 4 Lineberger Comprehensive Cancer Center, University of North Carolina School of Medicine, Chapel Hill, 27599-3280, NC, USA

**Keywords:** meiosis, recombination, cross-over, recombination variation

## Abstract

Most sexually reproducing eukaryotes use a specialized cell division called meiosis to halve the complement of chromosomes in their gametes. During meiotic prophase I, homologous chromosomes (homologs) recombine by reciprocally exchanging DNA to form cross-overs (COs) that are required for accurate chromosome segregation. COs also reshuffle parental genomes to create genetic diversity among progeny. Molecular genetic studies have identified hundreds of genes involved in meiotic recombination, which have been well summarized in several reviews. Here, we highlight recent advances in understanding endogenous mechanisms that regulate the frequency and distribution of meiotic COs, also called CO patterning. Specifically, we focus on genome-wide regulation, epigenetic control, transcription regulation, and post-transcription processes. Additionally, we highlight open questions that still need further investigation in this field.

## Introduction

Meiotic recombination is a key event during prophase I of meiosis. The reciprocal exchange of DNA, or COs, between homologous chromosomes (homologs) that results from recombination creates genetic diversity that is essential for biological evolution. It also establishes physical connections between homologs that ensure proper chromosome segregation. Disruption of meiotic recombination can cause infertility in both animals and plants. Conversely, the prospect of influencing the regulation of meiotic recombination through bioengineering holds great potential for plant and animal breeding and for human health.

 Meiotic recombination is initiated by DNA double-strand breaks (DSBs) in the DNA [1]. DSBs can be repaired using multiple pathways resulting in either COs or repair without reciprocal exchange of DNA, called non-cross-overs (NCOs). The frequency and distribution of meiotic COs are tightly regulated. Most species have a characteristic genome-wide number of COs with each pair of homologs having one to a handful, though there are notably exceptional species, such as the human fungal pathogen *Aspergillus fumigatus*, the honey bee *Apis mellifera,* and fission yeast *Schizosaccharomyces pombe* that have very high meiotic CO frequencies [2–4]. Typically, to ensure balanced chromosome segregation, each pair of homologs experiences at least one CO, called the obligate CO^5^. Another common phenomenon that regulates the patterning of COs is CO interference, which influences, usually by inhibiting, the formation of closely spaced double COs [5]. Most eukaryotes have two kinds of COs: type I, which are interference sensitive, and type II, which are interference insensitive. In most species, type I COs are more numerous and provide the obligate CO on each pair of homologs, while type II COs are less abundant and more randomly distributed. In many species, the number of meiotic DSBs is much greater than the ultimate number of COs, with the remainder of DSBs being repaired as NCOs. Interestingly, genetic manipulations or exposure to DNA-damaging agents, like ionizing radiation, that alter the number of DSBs within a certain range do not typically affect the number of COs but lead to changes in the number of NCOs, a phenomenon known as CO homeostasis, which has been best described in yeast [6]. Studies in *Arabidopsis thaliana* and *Zea mays* suggest that the CO homeostasis may be weaker in plants [7,8]. CO patterning is also regulated by regional or local factors including proximity to chromosome features, as is observed with suppression of COs near centromeres or the influence of DNA sequence motifs [9,10]. The degree to which these phenomena regulate CO patterning in different plant species, including crops, and how they might be mechanistically related largely remain open for exploration.

## Hotspots for initiation of meiotic recombination

**Figure 1:**
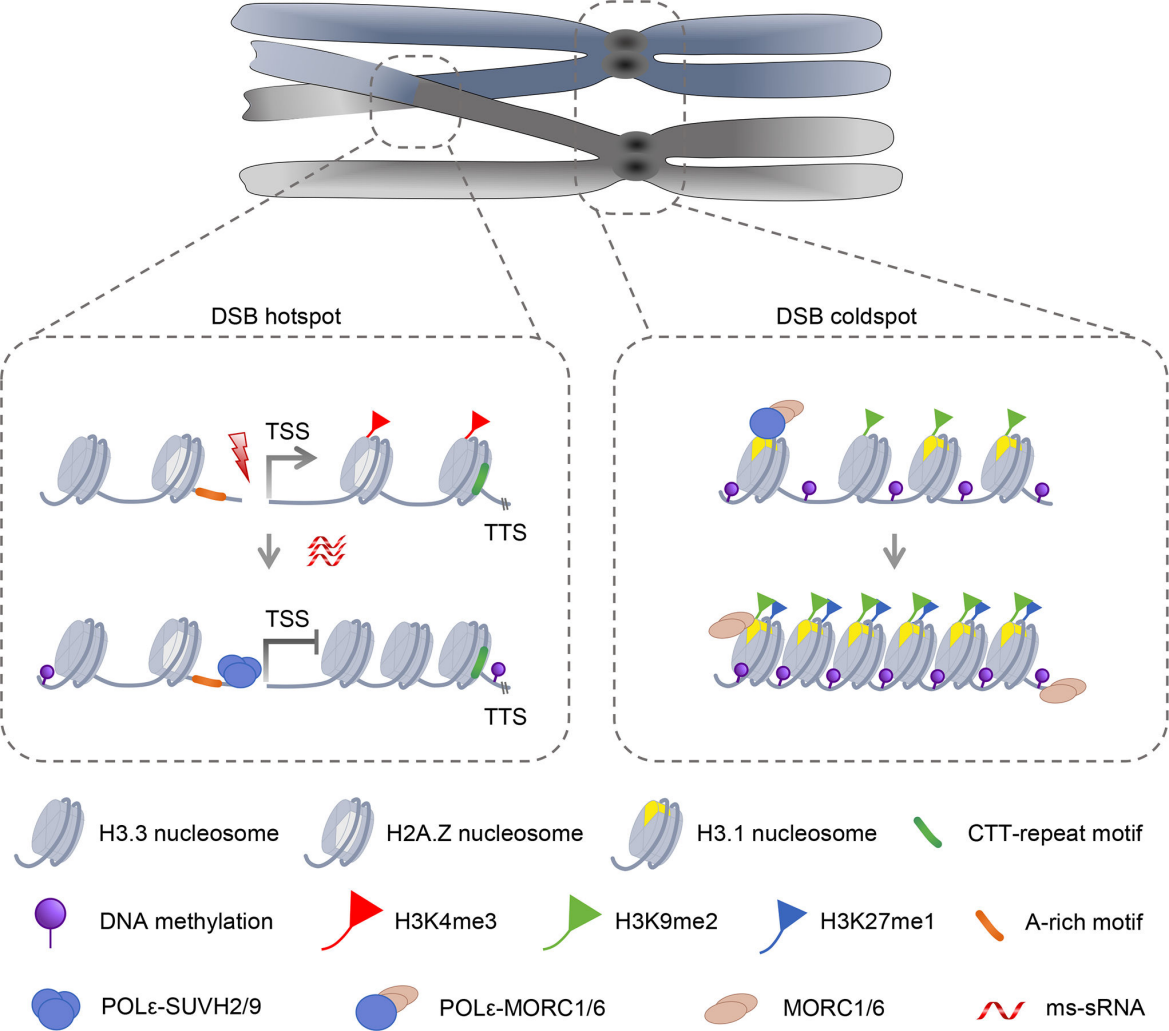
A schematic model for regulating hotspots for the initiation of meiotic recombination by epigenetic and sequence-based features in plants. DSB hotspots are associate with histone variant H3.3, H2AZ, CTT-repeat and A-rich motifs, H3K4me3, ms-sRNA, and low DNA methylation. DNA coldspots are associate with histone variant H3.1, H3K9me2, H3K27me1, and high DNA methylation. POLε recruit SUVH2 and SUVH9 to suppress gene transcription at meiotic DSB sites. POLε interacts with MORC1 and functions with MORC6 to organize meiotic heterochromatin condensation. DSB, double-strand break.

Meiotic DSBs are not uniformly distributed along chromosomes; instead, they occur mostly in euchromatin and rarely in heterochromatin and are typically clustered in regions called hotspots that span several kilobases [11]. Genomic and epigenetic characteristics such as DNA sequence motifs and chromatin modifications are pivotal factors for determining distribution of DSBs [12]. DSBs tend to occur at nucleosome-depleted regions adjacent to sites marked by H3K4me3 and H3K36me3 [13] that are established by PRDM9 in most mammalian species [14]. Plants do not appear to have PRDM9 homologs, but CO hotspots in plants are closely associated with three CTT-repeat and A-rich sequence motifs, as well as histone variant H2A.Z, histone H3K4me3, low nucleosome density, and low levels of DNA methylation (Figure 1) [1,15], all of which are conserved in plants.

Is there a connection between transcriptional activity and recombination in plants? In human somatic cells, DNA damage that results in DSBs adjacent to transcriptionally active regions has been observed to trigger transient transcriptional silencing [16]. The function of this phenomenon is unclear, but it is possible that transcriptional silencing at DSB sites might prevent aberrant recombination. Meiotic DSBs are also enriched in nucleosome-depleted, accessible regions with epigenetic markers for open chromatin [17], but whether transcriptional regulation at meiotic DSB influences CO patterning is largely an open question. In *Arabidopsis*, SPO11-1-dependent meiocyte-specific small RNAs (ms-sRNAs), which are likely a class of siRNA, are significantly enriched in coding regions and associated with genes that are preferentially expressed in meiosis (Figure 1) [18]. Additionally, SPO11-1-dependent sRNAs are associated with CTT-repeat and A-rich motifs that are meiotic CO hotspots, raising the possibility that ms-sRNA may be involved in regulating meiotic gene expression and recombination, thereby influencing CO patterning [18]. In addition, POL2A, the catalytic subunit of DNA polymerase epsilon (POLε), regulates CO patterning and transcription, in a role separate from its replication functions. POL2A recruits the SU(VAR)3 to 9 homologs SUVH2 and SUVH9 to suppress transcription at genes that are highly associated with DSB hotspots (Figure 1) [19]. Furthermore, two heterochromatin marks, H3K27me1 and H3K9me2, are reduced in *pol2a,* indicating that POL2A is also involved in organization of meiotic heterochromatin in DSB cold spots [20]. The C-terminal zinc finger domain of POL2A is specifically required for the deposition of histone variant H3.1–H4, which is essential for meiotic heterochromatin condensation, rather than H3.3 which is enriched in euchromatin [20]. POL2A also interacts, through its N terminus, with MORC1 (Microrchidia 1) ATPase, which together with MORC6 is required for meiotic heterochromatin condensation, indicating that distinct domains of POL2A synergistically organize meiotic heterochromatin condensation (Figure 1) [20].

## Transcriptional regulation

Genes that encode components of the meiotic recombination machinery are subject to transcriptional regulation. An important set of these components is the ZMM proteins, which promote COs by stabilizing recombination intermediates [21]. Among the ZMMs, the E3 ubiquitin ligase Human Enhancer of Invasion 10 (HEI10) acts as a dosage-sensitive regulator that controls the number and distribution of type I COs, which makes it a major target of CO frequency-regulating mechanisms [22]. Several type I CO regulators have been identified in *Arabidopsis* designated as high cross-over rates (HCRs) that control meiotic recombination by regulating HEI10 at transcriptional and post-translational levels. HCR2/heat shock factor binding protein (HSBP) represses *HEI10* transcription by directly binding heat shock transcription factors (HSFs) and thereby preventing their association with the *HEI10* promoter. In addition, HCR2/HSBP represses *HEI10* transcription through a second mechanism by maintaining hypermethylation at its 5′UTR. Through both mechanisms, repression of *HEI10* transcription leads to restricted CO numbers [23].

CO patterning may also be influenced by the transcriptional regulation of a wide range of recombination genes, rather than individual genes. *Arabidopsis* H3K9 demethylases Increase in Bonsai Methylation1 (IBM1)/JMJ25 and JMJ27 co-ordinately regulate meiotic recombination via their histone demethylase activity to prevent aberrant H3K9me2 at several meiotic loci and also regulate meiotic gene expression independently from their histone demethylase activity by interacting with homologs of the cohesin cofactor PDS5 [24,25]. Another transcriptional regulator of meiotic recombination gene expression is TAF4b (TBP-associated factor 4b), a subunit of the transcription factor TFIID. In the TFIID complex, TAF4b influences CO patterning by regulating transcriptional expression of meiotic genes, including several genes involved in recombination [26].

## Post-translational modifications

**Figure 2:**
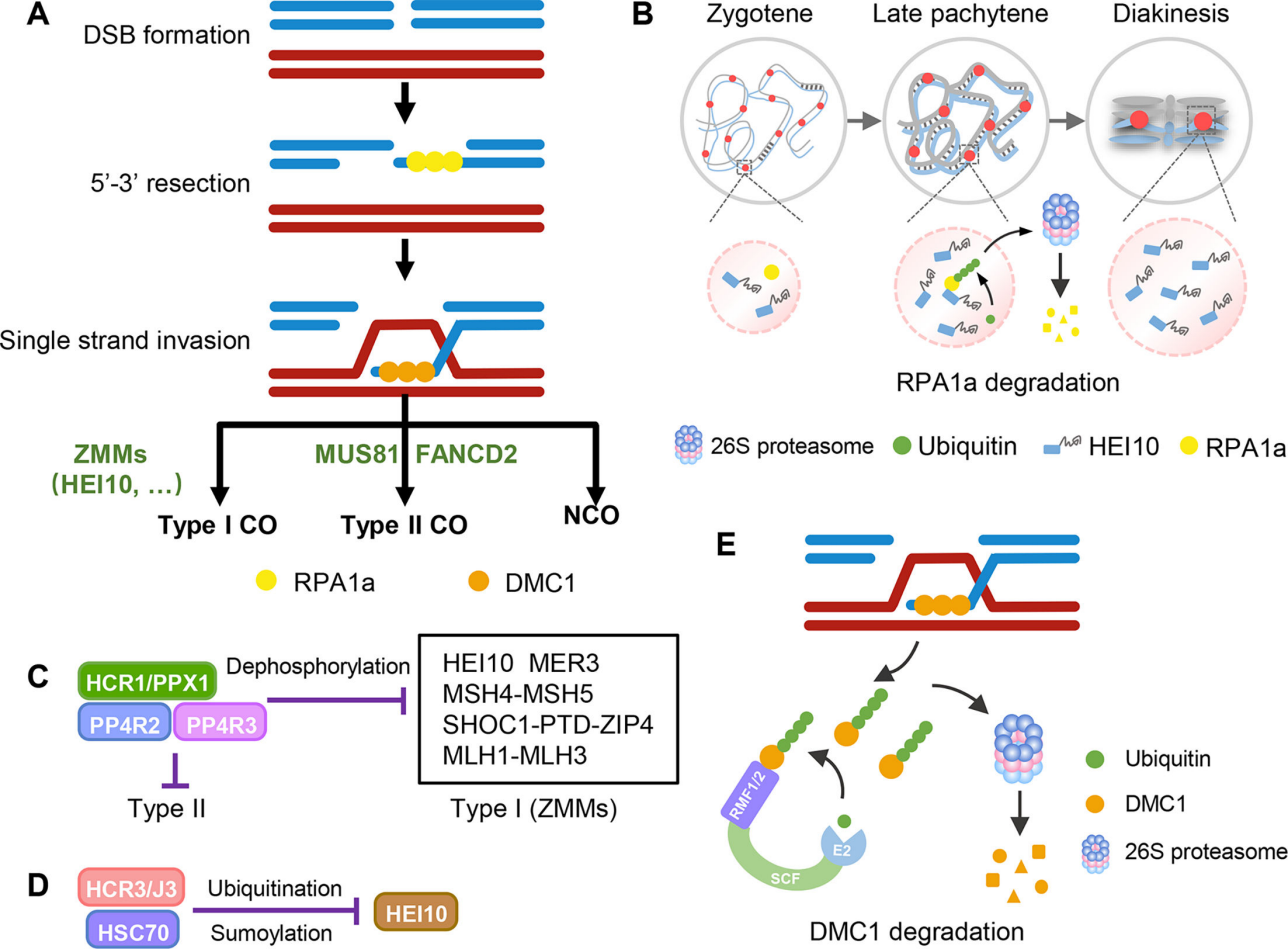
Schematic models for regulating meiotic cross-over (CO) by post-translational modification in plants. (**A**) A simplified model of meiotic recombination. It starts from the formation of the programmed double-strand breaks (DSBs). DSB ends are resected to yield 3’ overhangs of single-strand DNA (ssDNA) tails. RPA1a binds to 3’ssDNA to protect them from degradation. The subsequent replacement of RPA1a by RecA-related recombinases including DMC1 facilitates the invasion of the ssDNA into homologous non-sister chromatids to form a recombination intermediate called D-loop. Intermediates are resolved as ZMM protein-dependent interference sensitive (Type I) COs, MUS81 and FANCD2-dependent interference insensitive (Type II) COs, and non-cross-overs (NCOs) which are produced by synthesis-dependent strand annealing (SDSA) pathway. (**B**) A working model for HEI10’s phase separation and RPA1a proteolysis determining meiotic type I CO formation. Upper: Hundreds of HEI10 foci (magenta circle) localize along chromosome during zygotene and condense as larger foci at CO sites in pachytene and diakinesis. Bottom: HEI10 undergo liquid-liquid phase separation (LLPS) within the droplet during meiotic prophase I. In addition, HEI10 interacts with RPA1a to facilitate its ubiquitination and proteasomal degradation. (**C**) HCR1/PPX1 acts with PP4R2 and PP4R3 in PP4 protein phosphatase complex that might be responsible for dephosphorylation of HEI10 and other type I CO factors to antagonize type I CO formation, with alternative role in repressing type II CO. (**D**) A working model for HCR3/J3 and HSC70 involved in limiting COs by mediating the ubiquitination and SUMOylation of HEI10, thus promoting its proteasomal degradation. (**E**) A working model for ubiquitination of DMC1 by the SCF^RMF1/2^ complex during meiotic recombination. RMF1/2 interact with DMC1 and SCF^RMF1/2^ facilitates the ubiquitination of DMC1 for degradation by the 26S proteasome.

Post-translational modifications have been implicated in regulating meiotic recombination, and several post-translational modification factors that function in recombination have been recently identified. In mammals, putative SUMO ligase Ring Finger Protein 212 (RNF 212) and E3 ubiquitin ligase HEI10 act co-ordinately to facilitate the turnover of key recombination factors [27,28]. Replication Protein A 1 a (RPA1a) is one of HEI10’s direct substrates for ubiquitination and proteasomal degradation during meiotic recombination (Figure 2A and B, bottom row) [29]. Additionally, HEI10 undergoes liquid–liquid phase separation (LLPS), which is required for its condensation and coarsening on chromatin and type ICO formation (Figure 2A and B, upper row) [29]. Interestingly, HEI10 is also regulated by post-translational modification. Another member of the HCR factors discussed earlier, HCR1/Protein Phosphatase X1 (PPX1) is a catalytic subunit of the nuclear PP4 protein phosphatase complex that acts with PP4R2 and PP4R3 and has been implicated in dephosphorylation of HEI10 and other type I CO factors, including PTD, MSH5, and MLH1. Dephosphorylation of these factors is thought to suppress type I CO formation and might also restrict type II CO numbers (Figure 2C) [30]. In addition, HCR3/J3, a co-chaperone related to HSP40, along with HSC70, mediates the ubiquitination and SUMOylation of HEI10, which promote its proteasomal degradation, thus regulating the meiotic recombination landscape (Figure 2D) [31]. Phosphorylation has also been implicated in regulating meiotic recombination. In *Arabidopsis*, two key DNA damage response (DDR) pathway kinases ataxia-telangiectasia mutated (ATM) and ataxia-telangiectasia and rad3-related (ATR) play diverse roles through phosphorylating different targets. Mutation of ATR causes global changes to CO patterning, which suggest that ATR acts as a CO regulator at early stage during meiosis [32]. ATM regulates the number of meiotic DSB and type II COs, although its relevant phosphorylation targets and regulatory mechanisms require further study [33].

In most eukaryotes, repair of meiotic DSBs through homologous recombination relies on two RecA-related recombinases, RAD51, which functions in both mitosis and meiosis [34], and meiosis-specific DMC1 [35]. RAD51 and DMC1 are critical for mediating single-end invasion during recombination, and their stability and protein turnover are stringently regulated. In mammals, RAD51 and DMC1 are ubiquitinated for degradation in a 26S proteasome-dependent manner [27]. Consistent with those findings, a pair of functionally redundant F-box proteins RMF1/2 were identified in *Arabidopsis* that are responsible for ubiquitination and degradation of meiotic-specific recombinase DMC1 and crucial for CO formation [36]. Mutation of both *RMF1* and *RMF2* leads to aberrant synapsis of homologs, resulting in univalents rather than paired bivalents, unequal chromosome segregation, and failure to form COs, which is consistent with the meiotic recombination defects observed in transgenic plants with non-ubiquitinated DMC1 (Figure 2A and E) [36]. These recent studies demonstrate that post-translational modification is an important regulatory mechanism in meiosis, but much is yet to be understood about how it influences the activity of many meiotic proteins.

## Genome-wide and local regulation

COs are unevenly distributed along chromosomes, creating a recombination landscape with distinct peaks and valleys [15,37–39]. The distribution of COs correlates with various (epi)genomic features, including proximity to centromeres, transposable elements, sequence divergence, and chromatin state such as accessibility and DNA methylation [39–41].

**Figure 3:**
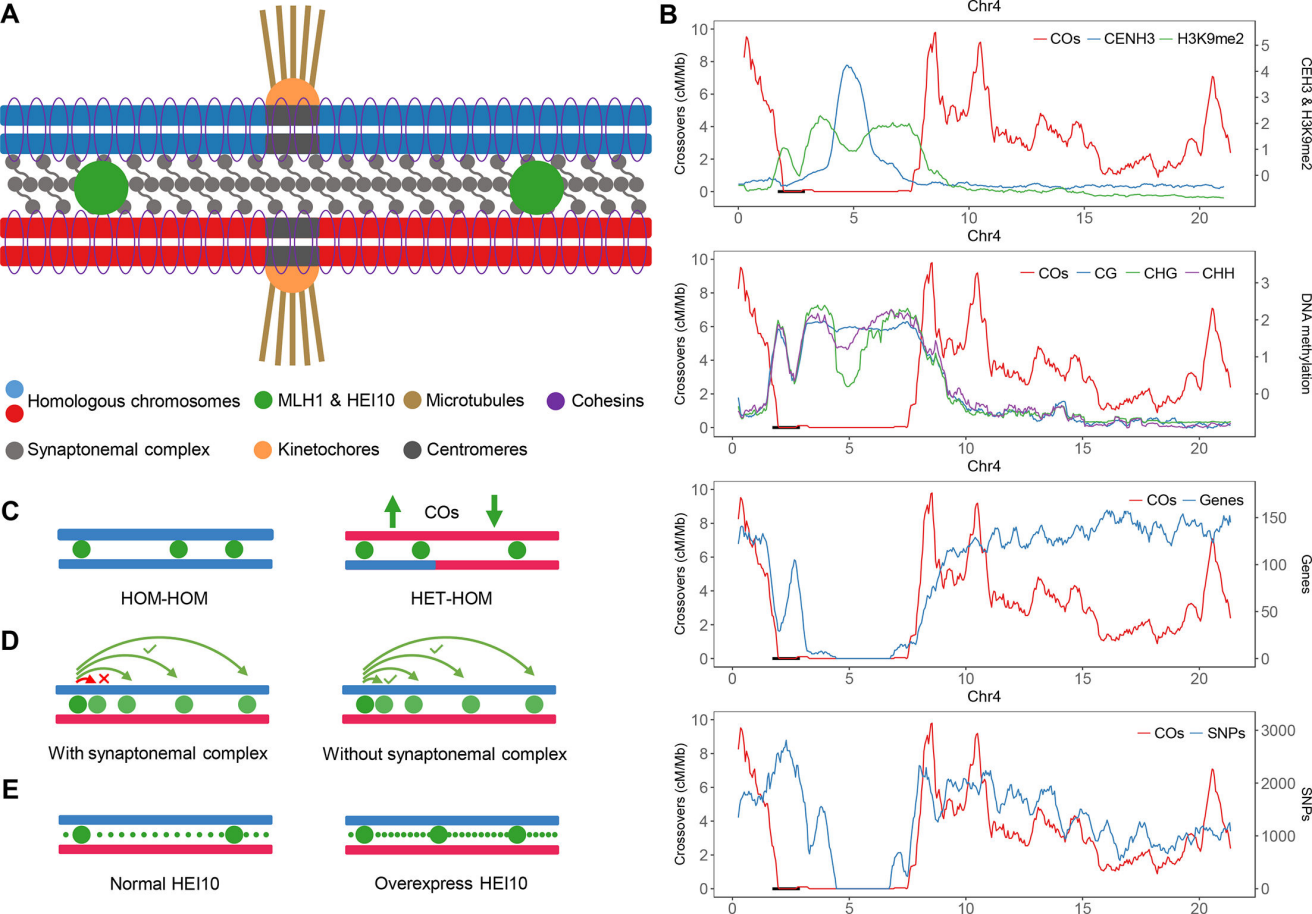
Genome-wide and local regulation of cross-over (CO) distribution. (**A**) A schematic representation of the chromosome at pachytene. Sister chromatids (blue and red) are tethered by cohesin rings (purple). Homologous chromosomes are joined by the synaptonemal complex (SC, gray). MLH1 and HEI10 mark the type I CO sites. Centromeres are labeled by black bars, where CENH3, kinetochores (orange) and spindle microtubules (brown) attached. (**B**) The chromosomal distribution along chromosome 4 (sliding window-based, window size 500 kb, step size 50 kb) of COs (red), CENH3 (blue), H3K9me2 (green), DNA methylation (blue, green and purple for CG, CHG and CHH), Genes (blue) and SNPs (blue) is plotted against the Col-CEN assembly (Naish et al., 2021 and Fernandes et al., 2024). The centromeric repeats was indicated by gray shading. The ~1.2 Mb inversion between col and L*er* on chromosome 4 is indicated by a black bar. (**C**) Schematic representation of the effect of juxtaposition of heterozygous and homozygous regions. The CO frequency in heterozygous regions is increased when juxtaposed with homozygous regions, which reciprocally decrease. (**D**) Schematic representation of the effect of synaptonemal complex. Disrupting the synaptonemal complex abolishes CO interference. (**E**) Schematic representation of the effect of HEI10 dosage. The overexpression of the pro-CO protein HEI10 increases COs but maintains CO interference.

Centromeres, the chromosomal regions where kinetochores form and spindle microtubules attach during cell division, are typically transposon-rich and enriched for histone H3 lysine 9 dimethylation (H3K9me2) and DNA methylation (Figure 3A–B) [42–44], as well as the histone variant H2A.W in *Arabidopsis* [45]. These regions exhibit considerable structural dynamics and divergence within and across species [46–49]. Recombination is generally suppressed at and near centromeres, a phenomenon known as the centromere effect, which is widely observed in eukaryotes [15,39,43,50,51]. Interestingly, the holocentric plant *Rhynchosporabreviuscula* has chromosomes with numerous diffuse centromeric domains, and COs are suppressed in each centromeric unit, but not nearby, indicating an absence of the classic centromere effect. The net result of diffuse centromeric units is a distally biased CO pattern [52]. The molecular mechanisms underlying pericentromeric CO repression remain poorly understood and likely vary between species. Studies in yeast show that the kinetochore and cohesin complexes inhibit the formation of meiotic DSBs and COs [50,51]. In *Arabidopsis*, H3K9me2 and DNA methylation repress both DSBs and COs in pericentromeric heterochromatin (Figure 3A–B) [42,53]. Also in *Arabidopsis*, the heterochromatin-specific histone variant H2A.W has recently been shown to suppress COs at pericentromeric heterochromatin [54,55].

Sequence polymorphisms can suppress CO formation locally and even abolish COs in large structural rearrangements, such as megabase-scale inversions (Figure 3B) [12,56–64]. However, in *Arabidopsis*, recombination rates in heterozygous regions increased when juxtaposed with homozygous regions (Figure 3C) [65]. In addition, the chromosomal recombination distribution in hybrids and natural populations often correlates strongly with polymorphisms. For instance, pericentromeric regions dense in polymorphisms also tend to have high CO rates (Figure 3B) [12,38,66]. Strikingly, a study in *Arabidopsis* found that the megabase-scale CO landscape is largely independent of sequence divergence [66]. Instead, chromatin features seem to play a more significant role in shaping the recombination landscape at both megabase and fine scales [66,67]. COs are also strongly associated with gene-dense regions along chromosomes, which are characterized by euchromatic features such as H2A.Z, H3K4me3, H3K36me3, low DNA methylation, and high chromatin accessibility [12,66,68,69]. In large-genome, TE-rich species such as barley and wheat, COs are strongly distal-biased, likely driven by early recombination initiation, homolog pairing, and synapsis at chromosome ends, where gene density, H3K27me3, and the chromosome axis protein ASY1 are enriched [69–72]. At a finer scale, COs tend to localize with gene promoters [66,67].

COs are also subject to CO interference (which has been recently reviewed extensively elsewhere), where type I COs inhibit the formation of additional nearby type I COs [73–75]. When CO interference is abolished, as in the absence of the synaptonemal complex (SC), COs tend to shift to more distal chromosomal regions (Figure 3D) [76–80]. However, although the interference-sensitive recombination increases in the zyp1 mutant, the observation of univalents implies a failure to form CO on each chromosome, which in turn implies a loss of CO assurance [76,81]. In fission yeast, which naturally lacks the SC, COs are essentially randomly distributed along chromosomes [4]. Furthermore, an emerging coarsening model suggests that HEI10 diffuses along the SC, controlling CO patterning in a dosage-dependent manner (Figure 3E) [77,82–85]. The coarsening model also provides a new perspective that CO interference is established by accumulating recombination factors at designated CO sites, thereby preventing the occurrence of other COs in the vicinity [82]. Interestingly, a recent study in *Arabidopsis* proposed that the CO landscape is shaped by the combination of chromatin state and sequence divergence, which determine the pool of eligible recombination intermediates, and the spatial organization of the SC, which influences CO designation [86].

In plants, CO patterning can also vary between male and female meiosis, a phenomenon known as heterochiasmy [87], which has in some cases been correlated with differences in the length of the SC or chromosome axis. In these instances, the CO rate usually increases proportionally with the length of the SC. A possible explanation for this observation is that if the CO interfere signal propagates along the SC and attenuates with distance, longer SCs will accommodate more COs [73]. Consistent with this idea, synapsis, interference, and heterochiasmy are all lost in absence of ZYP1 in *Arabidopsis* [76].

## Other mechanisms

In *Arabidopsis*, each meiosis generates 150–250 DSBs but only approximately 10 COs [88]. The remaining DSBs are repaired as NCOs or possibly through other mechanisms such as inter-sister repair, but the precise balance of those mechanisms has not been accurately measured in plants. The paucity of COs in such a large pool of DSBs suggests the activity of anti-CO factors. At least three anti-type II CO pathways have been discovered. First, the FANCM DNA helicase and its cofactors MHF1 and MHF2, which participate in NCO formation by unwinding D-loops as described in yeast [89,90]. Second, the members of the BTR (BLM-TOP3-RMI1) complex RECQ4A/4B DNA helicases, TOP3α, and RMI1 which, based on data from yeast, appear to promote NCO formation by resolving dHJs or D-loops [91]. Third, the AAA-ATPase FIGL1 and its interacting protein FLIP. Studies in *Arabidopsis* suggested that FIGL1 and FLIP antagonize CO formation by inhibiting dHJ-associated single-strand invasion during meiotic recombination [92]. In addition to their role in suppressing CO, these anti-CO factors affect CO patterning, as in *Arabidopsis*, FANCM, FIGL1, RMI1, along with FANCD2, act together to regulate the distribution of type I COs to enforce the formation of the obligate CO, but they play different roles in regulating the number of type II COs. FANCD2 promotes the formation of type II COs in *fancm* mutants but inhibits the formation of type II COs in *figl*1 and *rmi1* mutants [93].

In mammals, the majority of COs are type I, which are mediated by the ZMM proteins (ZIP1-4, MER3, and MSH4-5), as well as the MLH1 (MutL protein homologue 1)–MLH3 heterodimeric endonuclease (MutLγ) [88]. The remaining COs are type II and are mediated by MUS81 or FANCD2 [94,95]. In addition to these known CO formation proteins, HEIP1 (HEI10 Interacting Protein 1) was recently identified as a novel pro-CO factor in both rice and *Arabidopsis*, that is specifically required for type I CO formation and is conserved from plants to mammals [96,97].

Structural maintenance of chromosomes (SMC) proteins form complexes that have diverse roles in maintaining genome integrity. In addition to cohesin and condensin, which are both SMC complexes, SMC5/6 has roles in DNA replication and repair [98]. The SMC5/6 complex has been shown to have multiple essential roles in different steps in meiotic recombination. In *Arabidopsis*, SMC5/6 appears to antagonize the association of DMC1 with meiotic chromosomes, while RAD51 facilitates the action of DMC1 by inhibiting SMC5/6 so that DMC1 can bind to DSB sites [99], indicating the role of SMC5/6 in the early step of recombination. Intriguingly, QTL mapping of natural variants in *Arabidopsis* led to the identification of the SMC5/6 subunit SNI1 as a meiotic recombination modifier. Elevated COs in the distal regions and decreased COs in pericentromeric regions, as well as reduced CO interference, were observed in *sni1* mutant [100]. Consistent with these findings, mutations in other SMC5/6 subunits, including *nse2*, *nse4a*, *ASAP1/asap1* (*nse5*) heterozygotes and *smc6b*, show similar changes in meiotic recombination [100]. The mechanism by which SMC5/6 regulates CO patterning remain obscure, but one possibility is that SMC5/6 might prevent formation of aberrant recombination intermediates.

Polyploidy, which is common in plants, also influences recombination patterning. Allopolyploidization in several species, including *Arabidopsis* [101], *Brassica napus* [102], wheat [103], is accompanied by an increase in CO frequency. Studies in *Brassica* allotriploids showed increased CO frequencies that result from attenuated interference of type I COs, instead of an increase in type II COs [104,105]. In addition, several ZMM genes are differentially expressed in *Brassica* allotriploids [105]. Polyploidization can also affect CO distribution. In *Brassica napus*, odd ploidy disrupts the regulation of recombination, with COs occurring in pericentromeric regions [106], implying a dysregulation of the centromere effect.

Taken together, these recent findings, in multiple plant species, show that meiotic CO patterning is influenced by a variety of mechanisms. Some of these, such as the interplay of transcriptional regulation and recombination, need further investigation. Even when individual mechanisms become well characterized, it will be important to understand how they interact to form a cohesive regulatory network for enforcing the meiotic CO patterning we observe. With the development and application of advanced technologies, much remains to be done in the investigation of meiotic recombination.

**Perspectives**

- Meiotic cross-overs (COs) are required for accurate chromosome segregation and generate novel genetic combinations, driving diversity and evolution. The distribution of CO is tightly regulated by multiple regulatory layers, including genomic, epigenetic, transcriptional, and post-transcriptional mechanisms. How these regulatory mechanisms are co-ordinated to control the distribution of meiotic COs deserves further study.
- Numerous factors involved in meiotic recombination have been identified; however, the transcriptional and translational regulation of these factors, as well as the relationships between them, remains obscure. Chromatin modifications, DNA methylation, RNA modifications, and other emerging regulatory mechanisms in meiotic recombination require further investigation.
- While significant progress has been made in uncovering mechanisms that drive CO distribution in model species, research in crop species is rapidly advancing, leveraging cutting-edge technologies such as ultra-high-resolution and confocal microscopy, as well as whole-genome sequencing, with the goal of manipulating COs to accelerate plant breeding and harness hybrid vigor.

## Open Access

This article has been published open access under our Subscribe to Open programme, made possible through the support of our subscribing institutions, learn more here: https://portlandpress.com/pages/open_access_options_and_prices#conditional

## Abbreviations

COs: cross-overs
DSBs: double-strand breaks
LLPS: liquid–liquid phase separation
NCOs: non-cross-overs
SC: synaptonemal complex
SMC: Structural maintenance of chromosomes
ms-sRNAs: meiocyte-specific small RNAs
